# Supplementary material for: mRNA vaccine quality analysis using RNA sequencing
Source: Nat Commun. 2023 Sep 21;14:5663. doi: 10.1038/s41467-023-41354-y (PMC10514319; doi:10.1038/s41467-023-41354-y)
Supplement: Supplementary file 1 — Supplementary Information [file 41467_2023_41354_MOESM1_ESM.pdf]

mRNA vaccine quality analysis using RNA sequencing.

Helen M. Gunter[1,2*], Senel Idrisoglu[1,2*], Swati Singh[1,2], Dae Jong Han[2], Emily Ariens[2], Jonathan Peters[2], Ted Wong[3], Seth W. Cheetham[1,2], Jun Xu[4], Subash Kumar Rai[4], Robert Feldman[5], Andy Herbert[5], Esteban Marcellin[1], Romain Tropee[2], Trent Munro[1,2] and Tim R. Mercer[1,2]

[1] Australian Institute for Bioengineering and Nanotechnology, University of Queensland, Australia
[2] BASE facility, University of Queensland, Australia
[3] Garvan Institute of Medical Research
[4] Genome Innovation Hub, University of Queensland, Australia
[5] COVID19 Vaccine Corporation Limited (CVC), Auckland, New Zealand
[*] These authors contributed equally.

Supplementary Figures 1-12

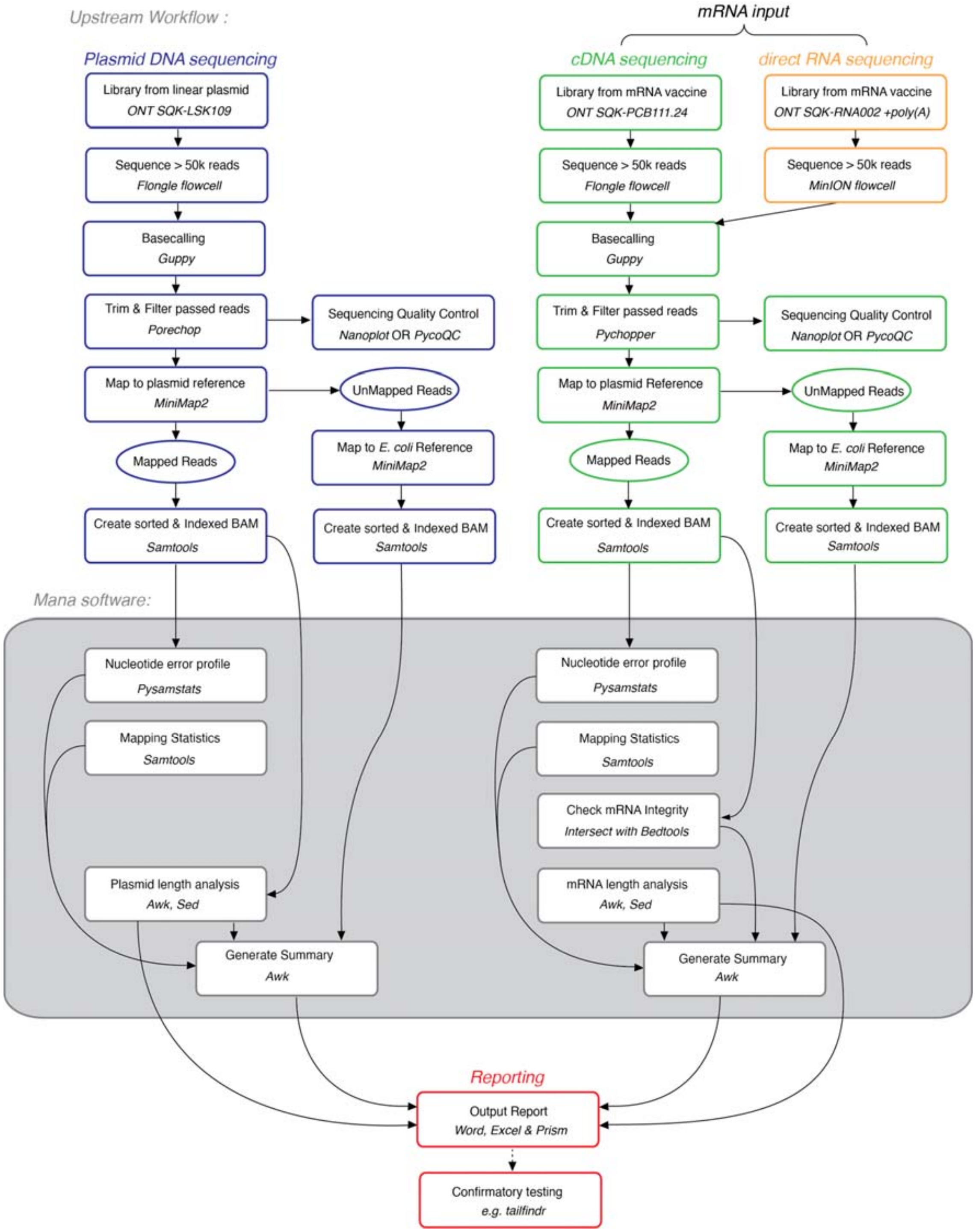

Supplementary Figure 1. VAX-seq pipeline, including laboratory, manual bioinformatic and *Mana* software analysis. Briefly, ONT libraries are prepared from linearised plasmid, cDNA and directly from mRNA, and are sequenced on either a Flongle or MinION flowcell. Reads are base-called, trimmed and aligned to the plasmid reference genome. Unmapped reads are aligned to the *E. coli* genome, to quantify any contamination from the plasmid preparation. Next, the *Mana* software utilises a range of software tools to compile a comprehensive report on the proportion of on target, truncated and extended mRNA transcripts. This information can be used to plan further analyses (such as *tailfindr*), optimise *in vitro* transcription workflows, and recommend whether an mRNA is suitable for use. Source data are provided as a Source Data file.

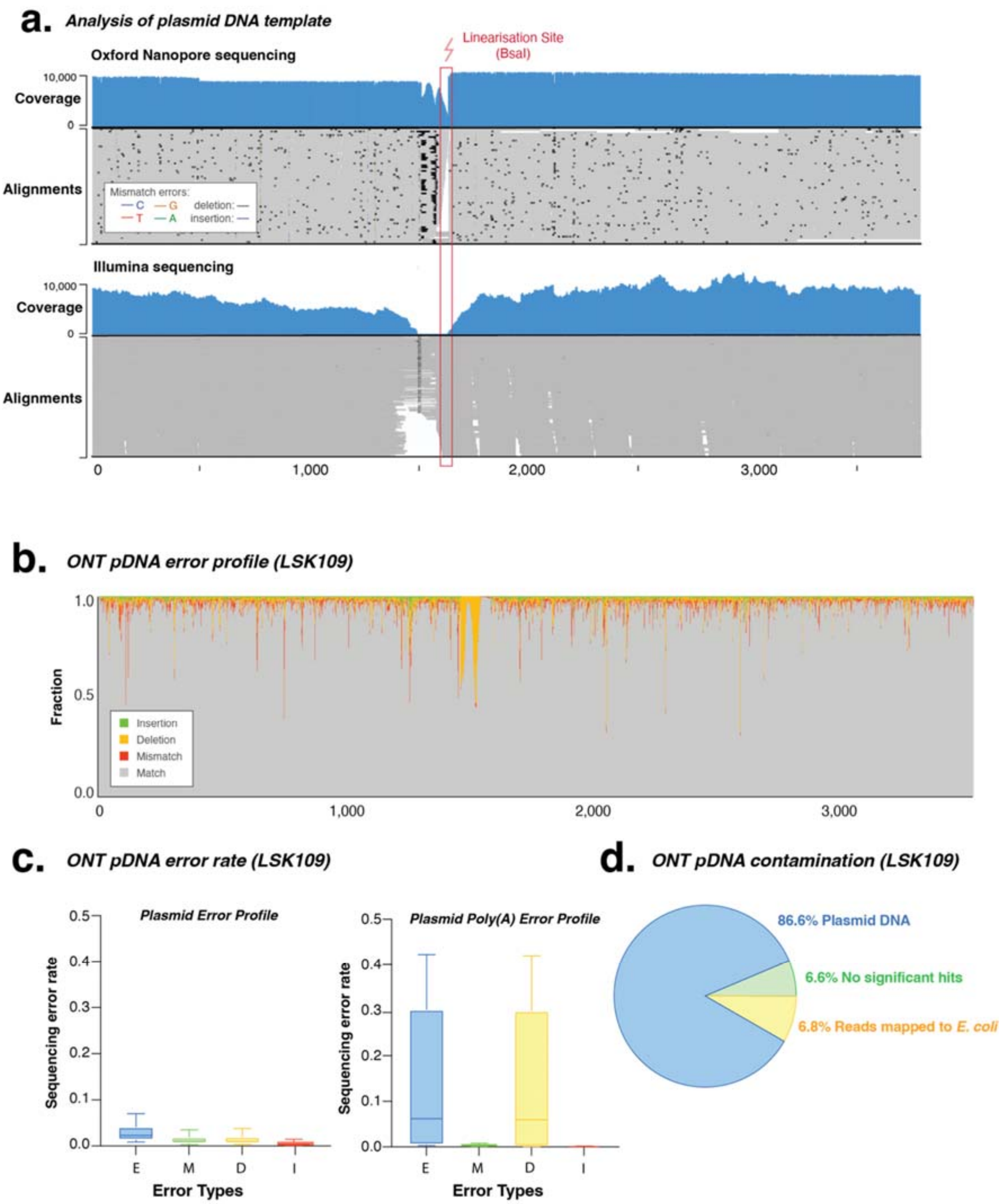

Supplementary Figure 2. Validation of Plasmid DNA quality using Oxford Nanopore Sequencing. (a) Genome-browser (IGV) view of long-read (ONT) and short-read (Illumina) alignments across sequenced plasmid DNA template, with linearisation site indicated (red box). Coverage indicates the number of reads at each nucleotide position whilst the lower alignments grey bars indicate unique, individual alignments, with colouring indicating their similarity to the reference genome. **(b)** Sequencing profile showing error type and frequency across the plasmid DNA template. **(c)** Box and whisker plots summarise the per nucleotide error rates within the mRNA vaccine and poly(A) tail sequence (E total errors, M mismatch, D deletion and I insertion). Box extends from 25th to 75th percentile, whiskers extend 10 – 90th percentile, centre plotted at median. Sequencing data derived from n=1 replicates. **(d)** Pie-chart shows the proportion of DNA contamination detected in linearised plasmid DNA preparations as measured using Oxford Nanopore sequencing. Source data are provided as a Source Data file.

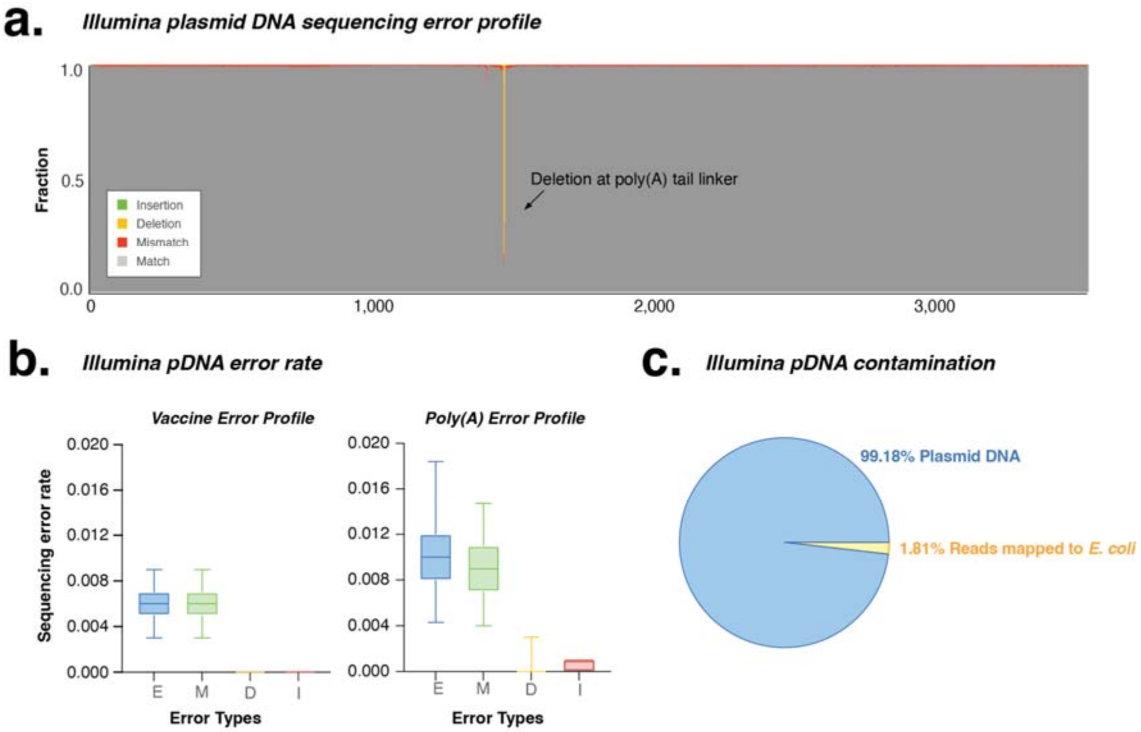

Supplementary Figure 3. Validation of plasmid DNA quality using short-read (Illumina) sequencing. (a) Sequencing profile shows error type and frequency across plasmid DNA template sequence. **(b)** Box and whisker plots show short-read sequencing per nucleotide error rates within the mRNA vaccine and poly(A) tail sequence. Box extends from 25th to 75th percentile, whiskers extend 10 – 90th percentile, centre plotted at median. Sequencing data derived from n=1 replicates. **(c)** Pie-chart illustrates the proportion of DNA contamination detected in plasmid sequencing libraries using Illumina sequencing. Source data are provided as a Source Data file.

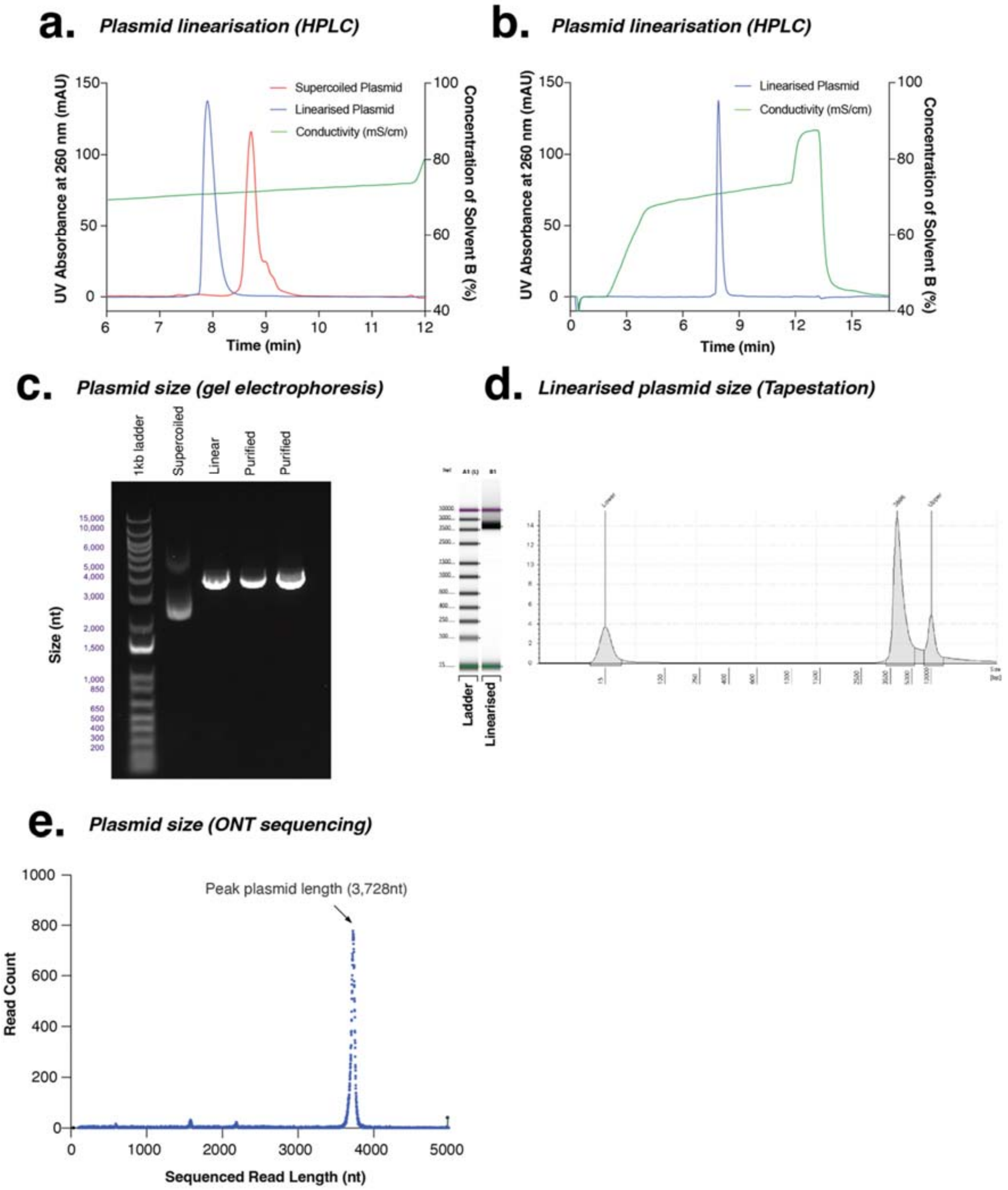

Supplementary Figure 4. Validation of plasmid length and linearisation. (a) High-performance liquid chromatography (HPLC) elution profile for coiled and linearized plasmids. **(b)** Linearisation efficiency as measured by HPLC analysis. **(c)** Agarose gel electrophoresis showing coiled, linearized and purified plasmid DNA preparations (n=1 supercoiled, n=1 linear, n=2 purified). **(d)** Analysis of linearized plasmid preparation using Agilent TapeStation shows size profile of plasmid preparations (n=1). **(e)** Size profile of linearised plasmid length as measured using long-read Oxford Nanopore sequencing. Source data are provided as a Source Data file.

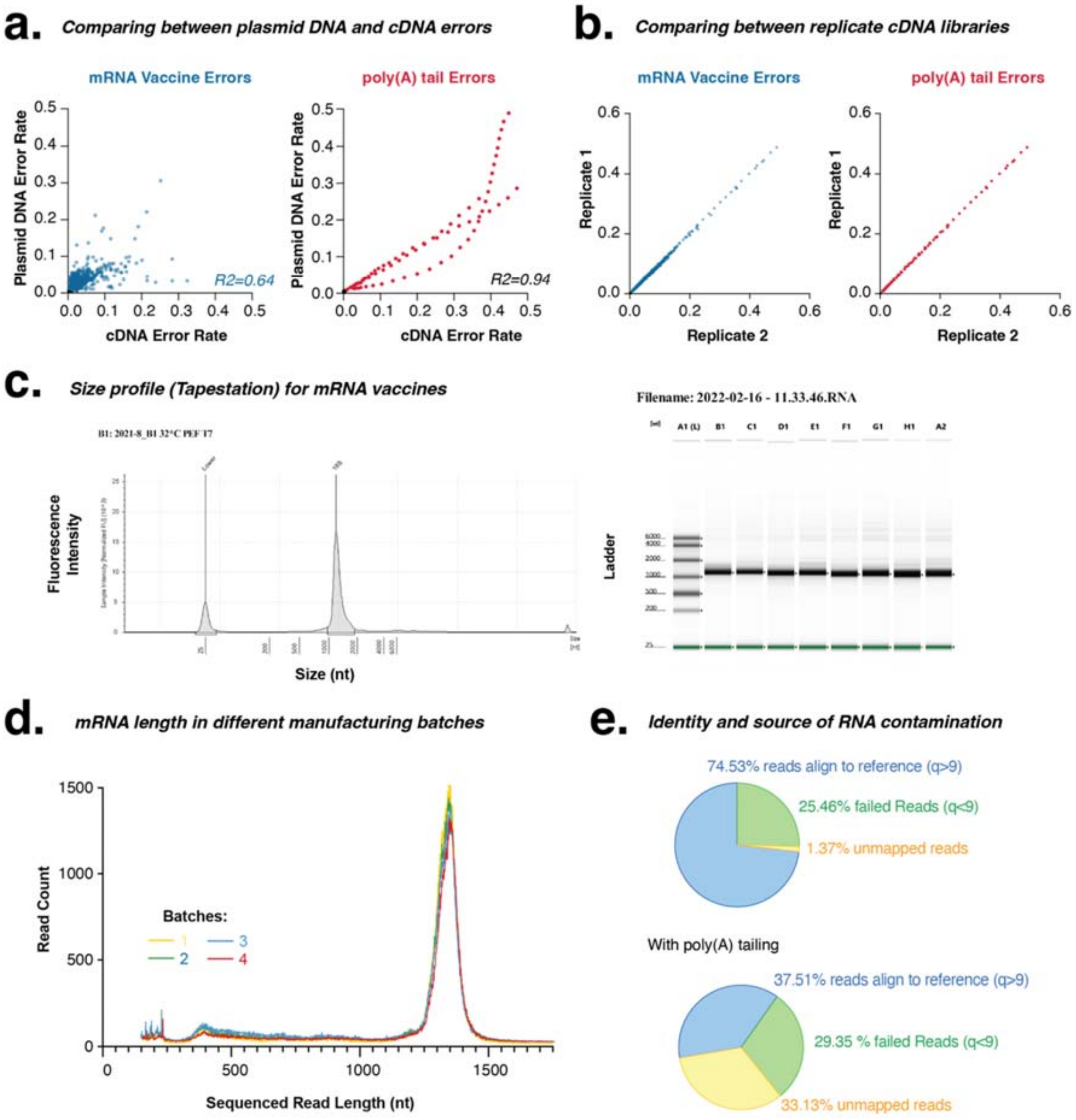

Supplementary Figure 5. Sequencing error rate. (a) Scatter-plot shows per-nucleotide comparison of error rate between cDNA and plasmid DNA sequencing within the mRNA vaccine sequence (blue) and poly(A) tail (red)**. (b)** Per-nucleotide pairwise comparison of sequencing error-rates between technical sequencing replicates in mRNA vaccine sequence (blue) and poly(A) tail (red). **(c)** Capillary electrophoresis size profile of reference eGFP mRNA vaccines (as measured with Agilent TapeStation) shows size profile (left) and capillary electrophoresis bands for replicate eGFP mRNAs transcribed from single plasmid preparation (right), (n=4). **(d)** Size plot generated from using long-read sequences shows the reproducibility for measuring mRNA length across multiple different manufacturing batches. **(e)** Pie chart shows the proportion of different contaminants as measured using long-read sequencing in mRNA vaccine samples (top) and samples that have undergone additional polyadenylation (bottom). Source data are provided as a Source Data file.

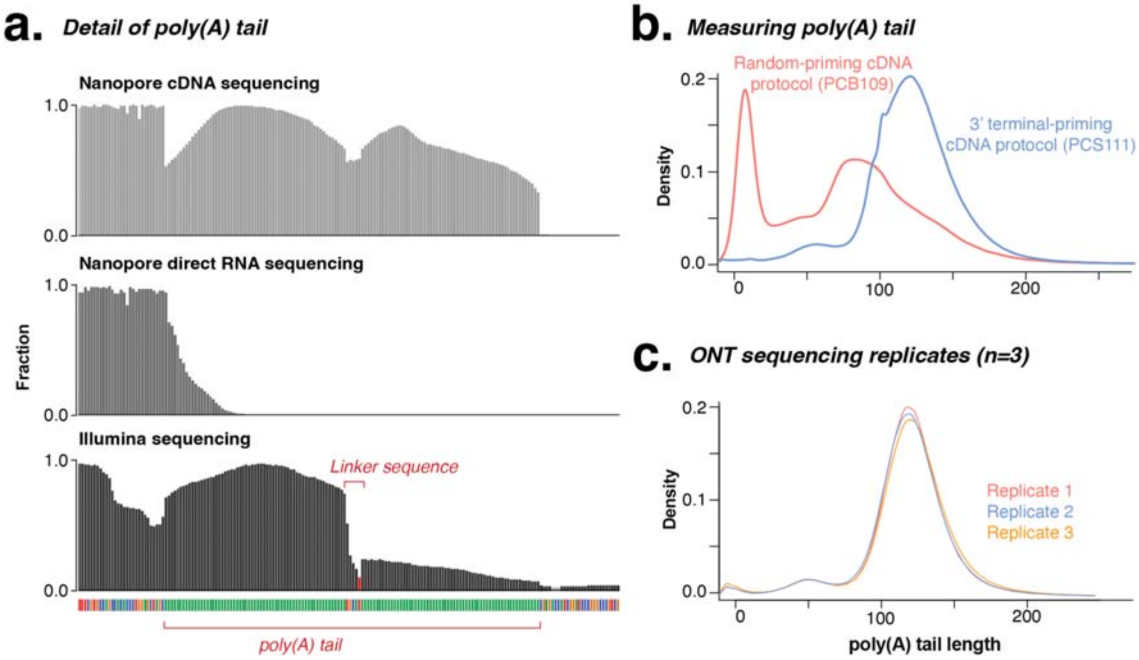

Supplementary Figure 6. Detail of poly(A) tail. (a) Detail shows alignment coverage at poly(A) tail for nanopore cDNA (top), direct RNA (middle) and Illumina (lower) sequencing. **(b)** Density plot shows the measurement of poly(A) tail length using *tailfindr* for the 3' terminal-priming cDNA (blue) and random-priming cDNA (red) sequencing protocols. **(c)** Density plot shows the reproducibility of measuring poly(A) tail length across technical replicates (n=3). Source data are provided as a Source Data file.

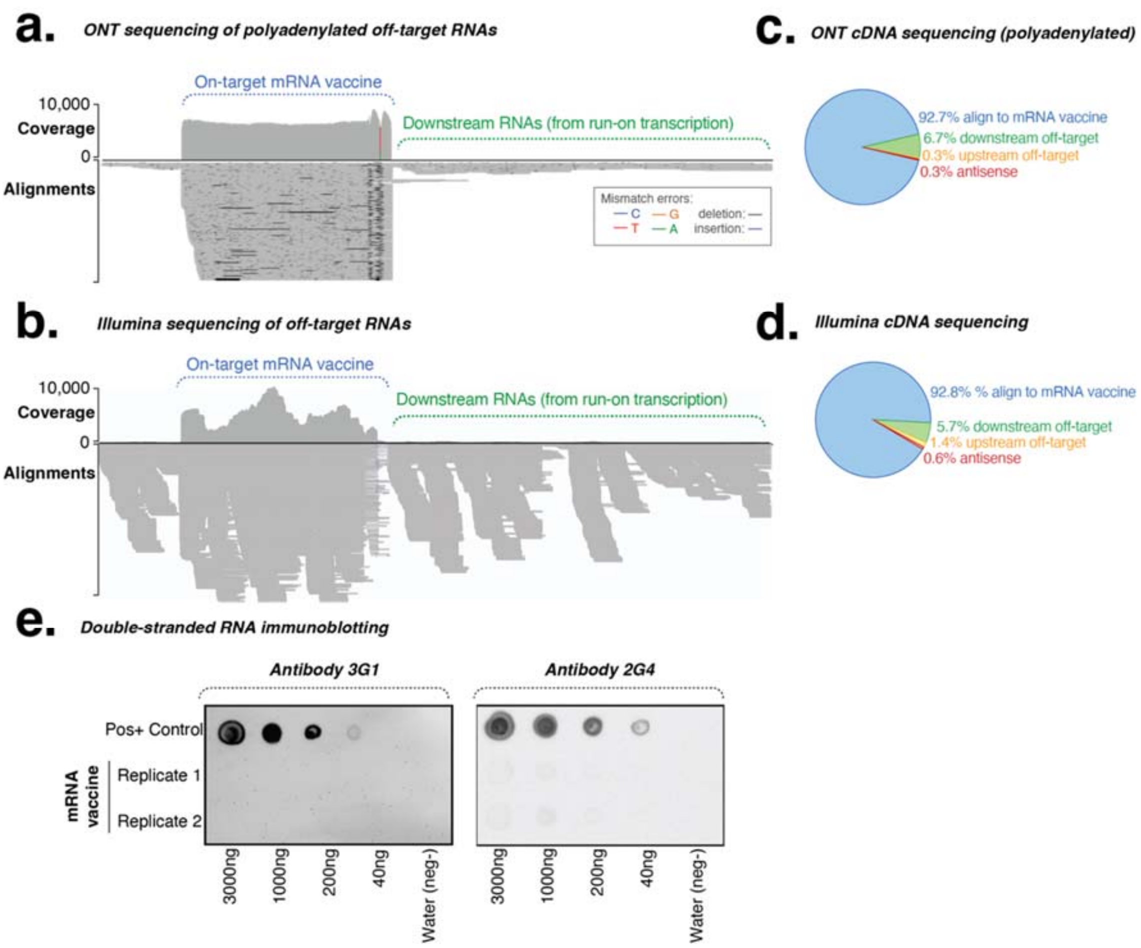

Supplementary Figure 7. Analysis of off-target RNA contamination. Genome browser view shows off-target RNA transcripts generated from cryptic transcription initiation or readthrough transcription for **(a)** long-read (following additional polyadenylation) or **(b)** short-read sequencing. Coverage indicates the number of reads at each nucleotide position whilst the lower alignments grey bars indicate unique, individual alignments, with colouring indicating their similarity to the reference genome. Pie-chart shows the composition of RNA contamination in mRNA samples as measured within **(c)** long- and **(d)** short-read sequencing libraries. **(e)** dsRNA immunoblotting using two different dsRNA-specific antibodies to detect the presence of double-stranded RNAs in mRNA vaccine samples. The dsRNA positive control was generated from previous manufacture[44]. Source data are provided as a Source Data file.

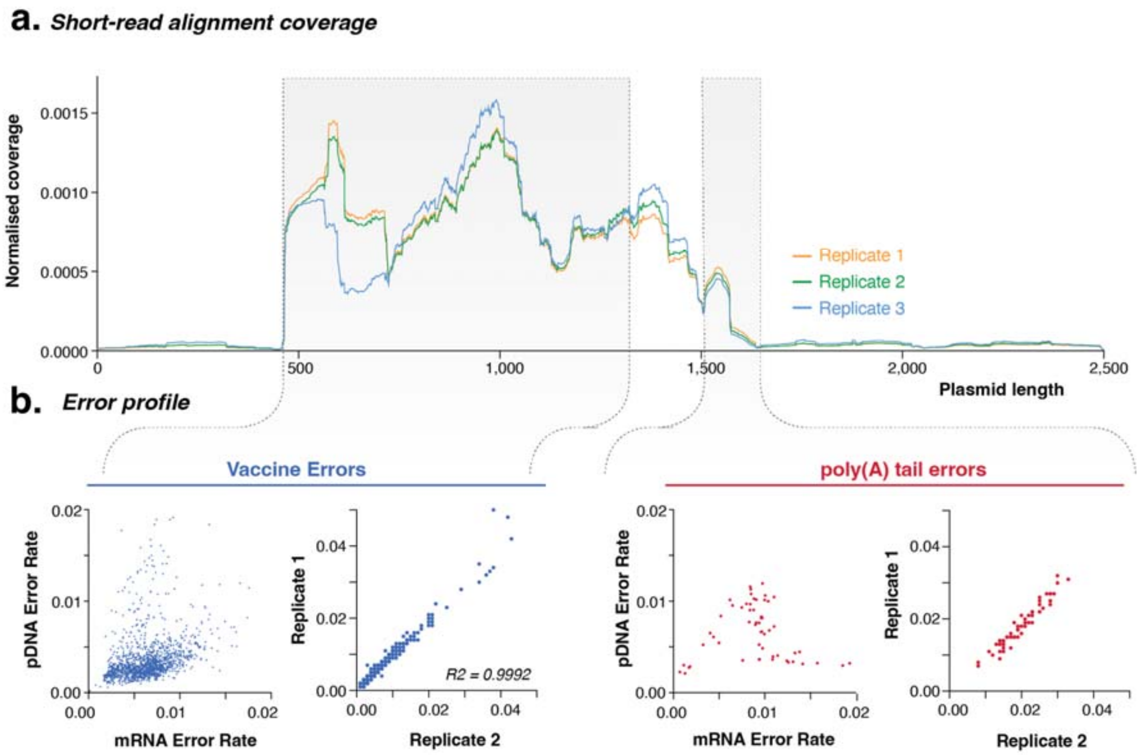

Supplementary Figure 8. Short-read Illumina sequencing of mRNA vaccine. (a) Histogram plot shows short-read alignment for an mRNA vaccine across replicate batches (n=3) showing reproducibility of alignment coverage. **(b)** Scatter plots compare per-nucleotide error rate in cDNA sequencing relative to plasmid DNA sequencing in an mRNA vaccine sequence (blue) and poly(A) tails (red), and per-nucleotide error frequency between replicate mRNA batches. Source data are provided as a Source Data file.

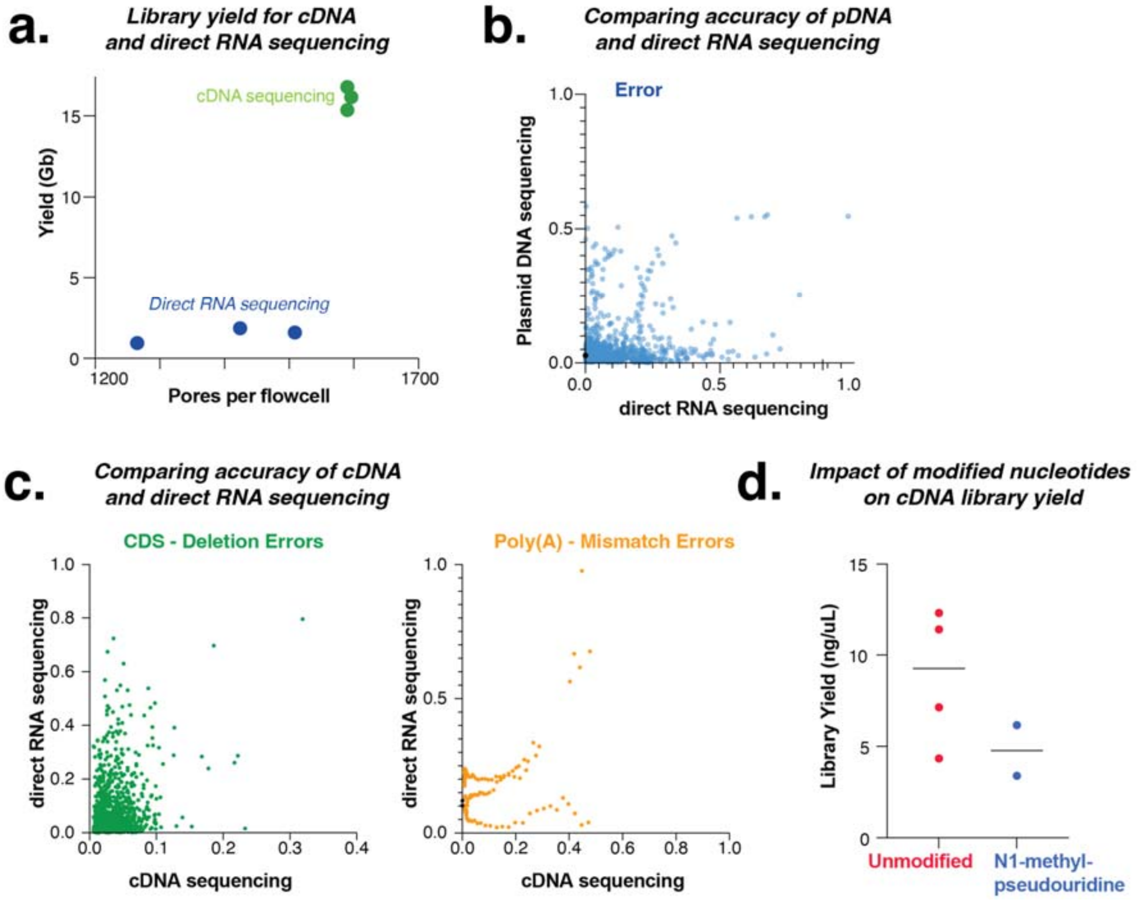

Supplementary Figure 9. Direct RNA sequencing of mRNA vaccines. (a) Scatter plot displaying the yield of libraries obtained from cDNA and direct RNA sequencing. **(b)** Scatter plot compares per-nucleotide error-rates between plasmid DNA and direct RNA sequencing in an mRNA vaccine. **(c)** Scatter plot compares per-nucleotide error rates between cDNA and direct RNA sequencing in the mRNA vaccine (green) and poly(A) tail (orange). **(d)** Comparison of cDNA library-yield between unmodified (red) and modified (blue; N1-methylpseudouridine) mRNA vaccines. (n=4 for unmodified libraries, and n=2 for modified libraries). Source data are provided as a Source Data file.

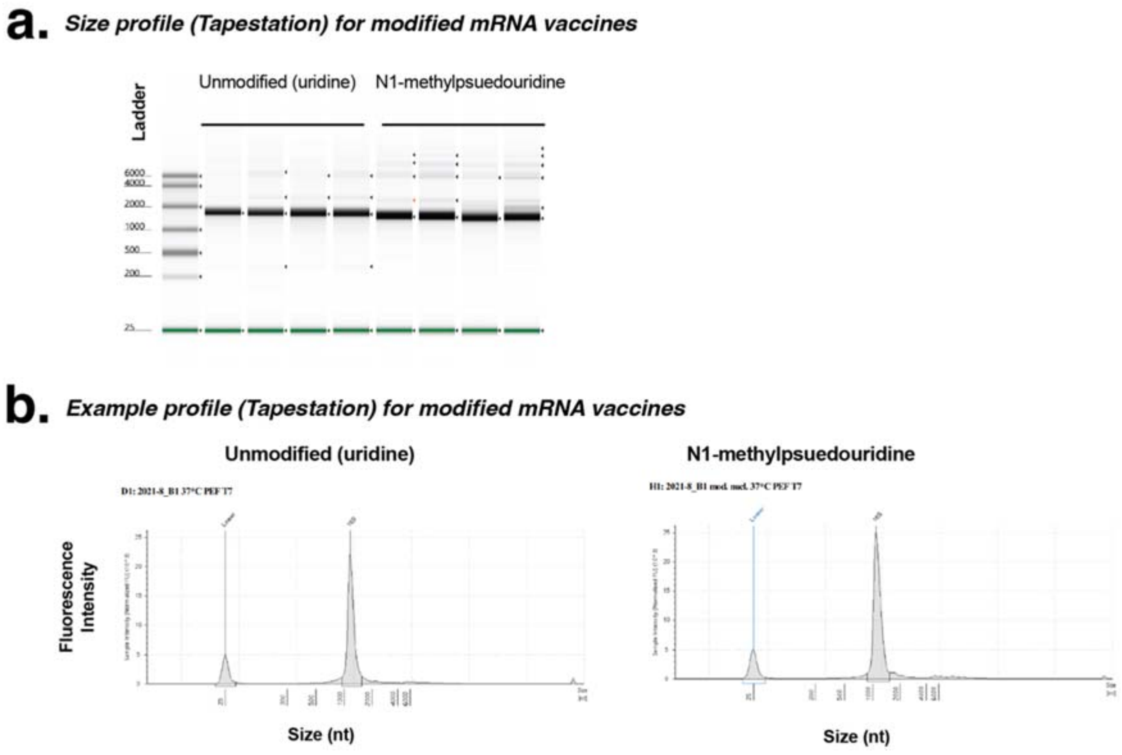

Supplementary Figure 10. Electrophoresis (Agilent TapeStation) size analysis of modified (N1-methylpseudouridine) mRNA vaccine. (a) Gel electrophoresis shows shorter migration of modified mRNA vaccines compared to unmodified mRNA vaccines in comparison to TapeStation RNA ladder (n=4). **(b)** Example size profiles for individual unmodified (left) and modified (right) mRNA vaccines. Source data are provided as a Source Data file.

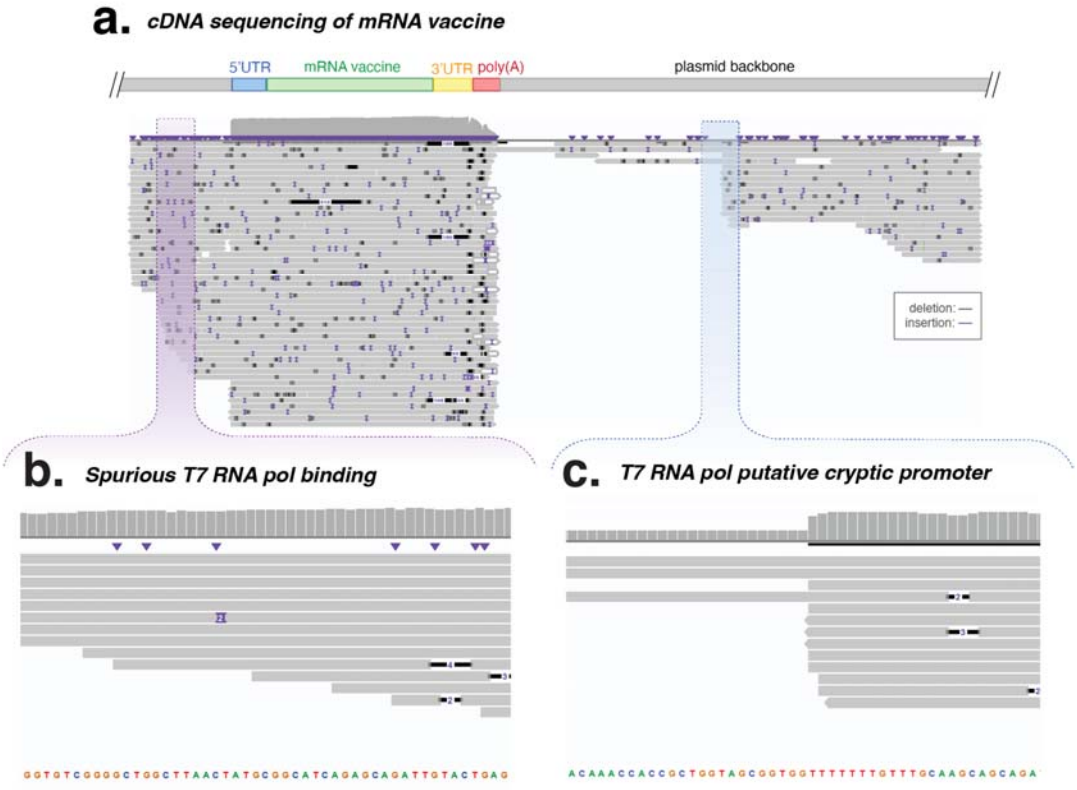

Supplementary Figure 11. Potential off-target binding of T7 RNA polymerase in an mRNA vaccine. (a) Genome browser view shows off-target transcripts identified by long-read sequencing of *in vitro* transcribed mRNA vaccine. **(b)** Off-target transcripts likely generated through spurious T7 RNA polymerase binding, 5' to the T7 promoter included in our construct. **(c)** Additionally, a potential cryptic T7 promoter was identified, where transcription appears to have been initiated at the same position for eight reads, potentially due to the presence of a polypyrimidine tract. T7 promoter binding affinity at this position could be tested by DNA foot-printing analyses. Source data are provided as a Source Data file.

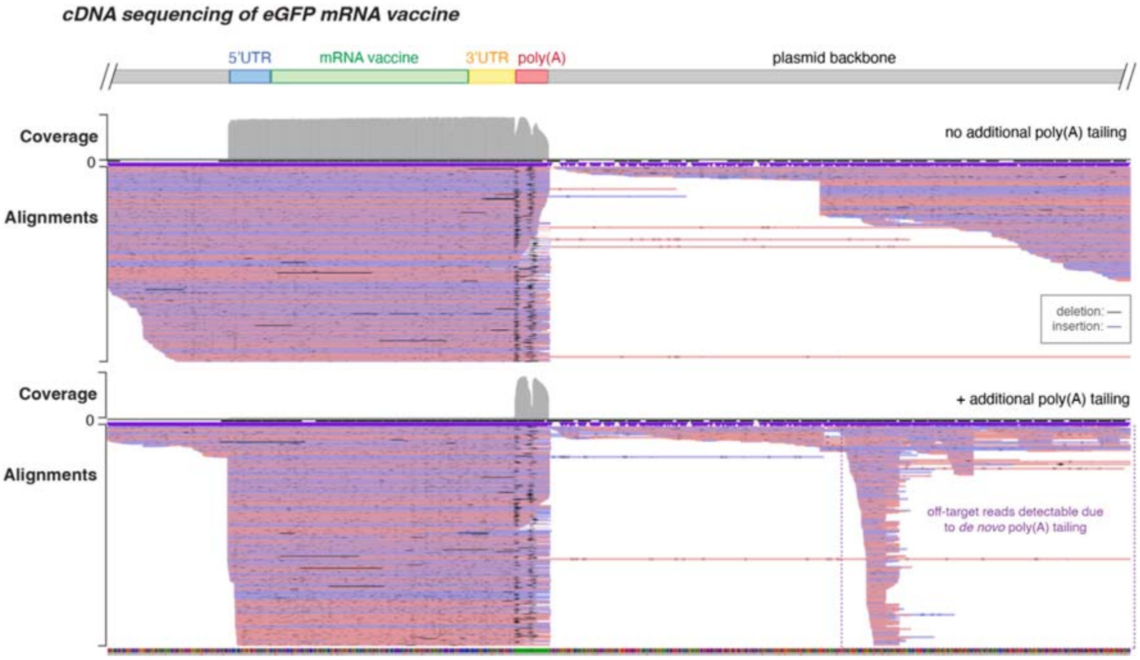

Supplementary Figure 12. ***De novo*** **poly(A) tailing of eGFP mRNA permits detection of additional off-target transcripts.** Genome browser view comparing ONT cDNA sequencing libraries that include additional poly(A) tailing (bottom panel) to libraries that lack additional poly(A) tailing (top panel). Additional off-target transcripts are observed in libraries with *de novo* poly(A) tailing in comparison to those without *de novo* poly(A) tailing, as cDNA sequencing libraries involve a poly(A) selection step. Source data are provided as a Source Data file.

Source Data files

Figure S4c

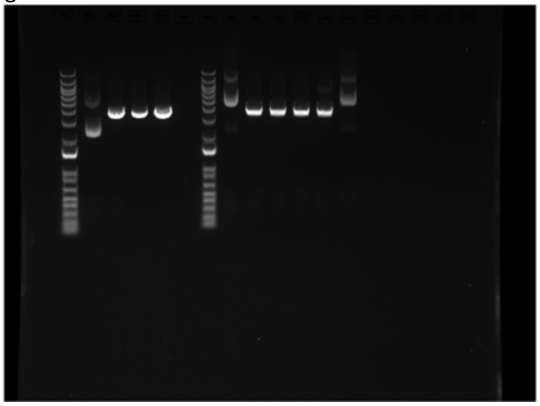

Figure S4d

Filename: 2022-05-03 - 16.18.40.D5000

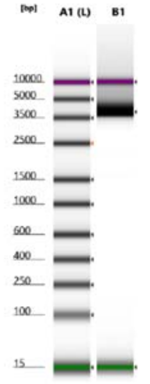

Default image (Contrast 100%)

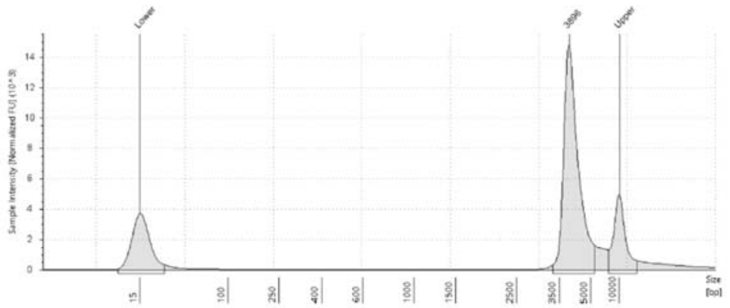

Sample Table

Well	Conc. [ng/µl]	Sample Description	Alert	Observations
B1	21.4	GFP LSK109 end rep		

Peak Table

Size [bp]	Calibrated Conc. [ng/µl]	Assigned Conc. [ng/µl]	Peak Molarity [nmol/l]	% of Total	% Integrated Area	Peak Comment	Observations
15	5.82	-	597	-	-		Lower Marker
3896	21.4	-	8.46	79.61	100.00		
10000	3.25	3.25	0.500	-	-		Upper Marker

Figure S5c, S10a

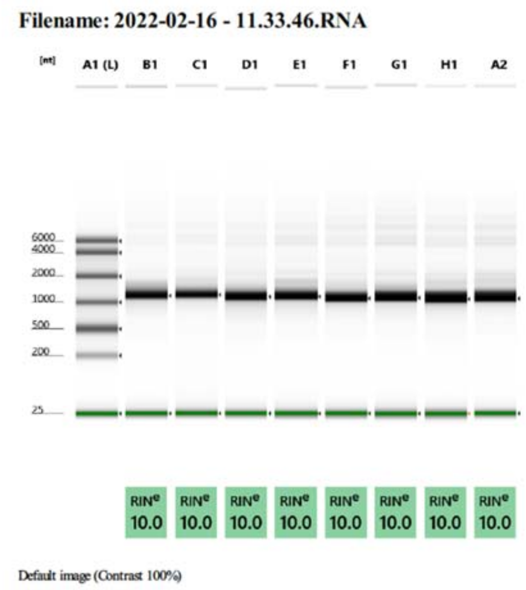

Figure S7e: 3G1 antibody

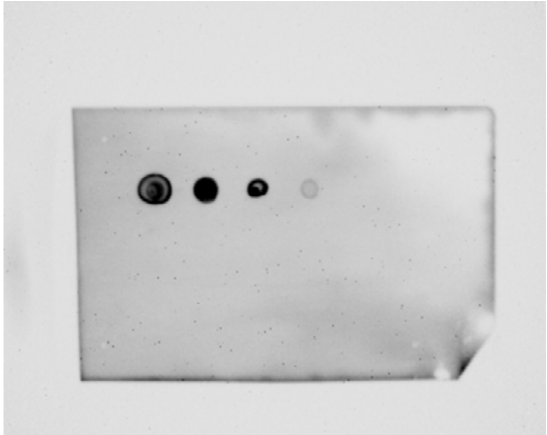

Figure S7e: 2G4 antibody

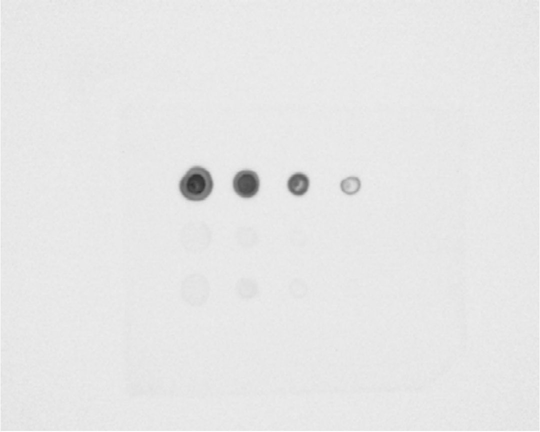

Figure S10b

D1: 2021-8_B1 37°C PEF T7

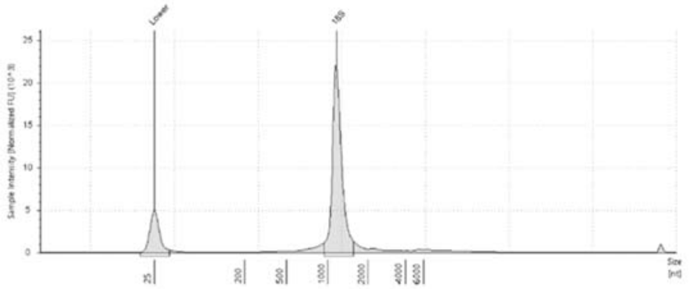

Sample Table

Well	RINe	28S/18S (Area)	Conc. [ng/µl]	Sample Description	Alert	Observations
D1	10.0	-	112	2021-8_B1 37°C PEF T7		The upper ribosomal fragment has degraded

Peak Table

Size [nt]	Calibrated Conc. [ng/µl]	Assigned Conc. [ng/µl]	Peak Molarity [nmol/l]	% Integrated Area	Peak Comment	Observations
25	36.0	36.0	4240	-		Lower Marker
1160	85.4	-	217	100.00		18S

H1: 2021-8_B1 mod. nucl. 37°C PEF T7

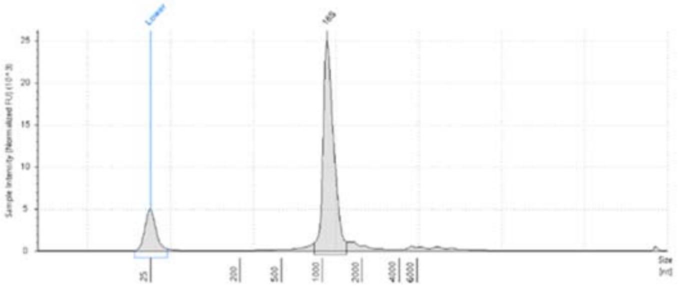

Sample Table

Well	RINe	28S/18S (Area)	Conc. [ng/µl]	Sample Description	Alert	Observations
H1	10.0	-	117	2021-8_B1 mod. nucl. 37°C PEF T7		The upper ribosomal fragment has degraded

Peak Table

Size [nt]	Calibrated Conc. [ng/µl]	Assigned Conc. [ng/µl]	Peak Molarity [nmol/l]	% Integrated Area	Peak Comment	Observations
25	36.0	36.0	4240	-		Lower Marker
1090	94.1	-	254	100.00		18S
