# Supplementary material for: mRNA vaccine quality analysis using RNA sequencing
Source: Nat Commun. 2023 Sep 21;14:5663. doi: 10.1038/s41467-023-41354-y (PMC10514319; doi:10.1038/s41467-023-41354-y)
Supplement: Supplementary file 3 — Reporting Summary [file 41467_2023_41354_MOESM3_ESM.pdf]

nature portfolio

Corresponding author(s): Tim Mercer

Last updated by author(s): 24.08.2023

Reporting Summary

Nature Portfolio wishes to improve the reproducibility of the work that we publish. This form provides structure for consistency and transparency in reporting. For further information on Nature Portfolio policies, see our Editorial Policies and the Editorial Policy Checklist.

Statistics

For all statistical analyses, confirm that the following items are present in the figure legend, table legend, main text, or Methods section.

n/a	Confirmed	
☐	☒	The exact sample size (*n*) for each experimental group/condition, given as a discrete number and unit of measurement
☐	☒	A statement on whether measurements were taken from distinct samples or whether the same sample was measured repeatedly
☒	☐	The statistical test(s) used AND whether they are one- or two-sided<br>*Only common tests should be described solely by name; describe more complex techniques in the Methods section.*
☒	☐	A description of all covariates tested
☒	☐	A description of any assumptions or corrections, such as tests of normality and adjustment for multiple comparisons
☒	☐	A full description of the statistical parameters including central tendency (e.g. means) or other basic estimates (e.g. regression coefficient) AND variation (e.g. standard deviation) or associated estimates of uncertainty (e.g. confidence intervals)
☒	☐	For null hypothesis testing, the test statistic (e.g. *F*, *t*, *r*) with confidence intervals, effect sizes, degrees of freedom and *P* value noted<br>*Give P values as exact values whenever suitable.*
☒	☐	For Bayesian analysis, information on the choice of priors and Markov chain Monte Carlo settings
☒	☐	For hierarchical and complex designs, identification of the appropriate level for tests and full reporting of outcomes
☒	☐	Estimates of effect sizes (e.g. Cohen's *d*, Pearson's *r*), indicating how they were calculated

Our web collection on statistics for biologists contains articles on many of the points above.

Software and code

Policy information about availability of computer code

Data collection	Guppy v5.1.13; MinKNOW Core 4.5.4
Data analysis	Minimap2 (2.20-r1064); SAMtools v1.15, Integrative Genomics Viewer (IGV v2.12.3), NanoPlot v1.38.1, pycoQC v v2.5.2, pysamstats v 1.1.2, bcftools, Excel v 16.67 for Mac, GraphPad Prism v9, Mana software, available through: https://github.com/scchess/Mana, Illumina DRAGEN BCL Convert 07.021.609.3.9.3 pipeline, TrimGalore (v0.6.8dev), FastQC, bwa-0.7.17-r1188, pychopper (v.2.5.0), BEDtools (v2.27.1), tailfindr (v1.3) , Porechop v0.2.4, Nanopolish v0.13.3.

For manuscripts utilizing custom algorithms or software that are central to the research but not yet described in published literature, software must be made available to editors and reviewers. We strongly encourage code deposition in a community repository (e.g. GitHub). See the Nature Portfolio guidelines for submitting code & software for further information.

Data

Policy information about availability of data

All manuscripts must include a data availability statement. This statement should provide the following information, where applicable:

- Accession codes, unique identifiers, or web links for publicly available datasets
- A description of any restrictions on data availability
- For clinical datasets or third party data, please ensure that the statement adheres to our policy

The genomic and transcriptomic data generated in this study have been deposited in the Sequence Read Archive (SRA) under accession codes: PRJNA856796 for

RNA-seq (https://www.ncbi.nlm.nih.gov/bioproject/PRJNA856796), and PRJNA854780 for plasmid re-sequencing (https://www.ncbi.nlm.nih.gov/bioproject/PRJNA854780). The E. coli genome is available through: https://www.ncbi.nlm.nih.gov/nuccore/NC_000913.3?report=fasta. Further data generated in this study are provided in the Source Data File, with larger files available through DRYAD (doi:10.5061/dryad.s1rn8pkds).

Research involving human participants, their data, or biological material

Policy information about studies with human participants or human data. See also policy information about sex, gender (identity/presentation), and sexual orientation and race, ethnicity and racism.

Reporting on sex and gender	N/A
Reporting on race, ethnicity, or other socially relevant groupings	N/A
Population characteristics	N/A
Recruitment	N/A
Ethics oversight	N/A

Note that full information on the approval of the study protocol must also be provided in the manuscript.

Field-specific reporting

Please select the one below that is the best fit for your research. If you are not sure, read the appropriate sections before making your selection.

- [x] Life sciences
- [ ] Behavioural & social sciences
- [ ] Ecological, evolutionary & environmental sciences

For a reference copy of the document with all sections, see nature.com/documents/nr-reporting-summary-flat.pdf

Life sciences study design

All studies must disclose on these points even when the disclosure is negative.

Sample size	No statistical methods were used to pre-determine sample size. Our data represent a proof of concept.
Data exclusions	No data were excluded.
Replication	Replication was used for four different analyses: (i) three analyses were n=3 and (ii) four analyses were n=4. All attempts at replication were successful.
Randomization	No randomisation was used in this manuscript. All libraries were prepared by a single person, to reduce operator-based biases. Flow cell based variation was controlled in the Illumina analyses, by sequencing all libraries on a single Flow Cell. For the Oxford Nanopore sequencing, Flow Cell variability was measured for the direct RNA sequencing replicates, which were run on separate flow cells. Limited variability was observed for these replicates.
Blinding	No blinding was used in this manuscript. We feel that knowledge of our construct design would not impact the interpretation of our results.

Reporting for specific materials, systems and methods

We require information from authors about some types of materials, experimental systems and methods used in many studies. Here, indicate whether each material, system or method listed is relevant to your study. If you are not sure if a list item applies to your research, read the appropriate section before selecting a response.

Materials & experimental systems

n/a	Involved in the study	
[ ]	[x]	Antibodies
[x]	[ ]	Eukaryotic cell lines
[x]	[ ]	Palaeontology and archaeology
[x]	[ ]	Animals and other organisms
[x]	[ ]	Clinical data
[x]	[ ]	Dual use research of concern
[x]	[ ]	Plants

Methods

n/a	Involved in the study	
[x]	[ ]	ChIP-seq
[x]	[ ]	Flow cytometry
[x]	[ ]	MRI-based neuroimaging

Antibodies

Antibodies used	murine monoclonal antibodies derived from clones 3G1 and 2G4 (Mozzy Mabs, Brisbane, Australia).
Validation	Validated with double stranded RNA described in Mitter, N. et al. Clay nanosheets for topical delivery of RNAi for sustained protection against plant viruses. Nat. Plants 3, 16207 (2017).
